# Microbial Interactions during Upper Respiratory Tract Infections

**DOI:** 10.3201/eid1410.080119

**Published:** 2008-10

**Authors:** Melinda M. Pettigrew, Janneane F. Gent, Krystal Revai, Janak A. Patel, Tasnee Chonmaitree

**Affiliations:** Yale School of Public Health, New Haven, Connecticut, USA (M. Pettigrew, J. Gent); University of Texas Medical Branch, Galveston, Texas, USA (K. Revai, J. Patel, T. Chonmaitree)

**Keywords:** Streptococcus pneumoniae, Haemophilus influenzae, Moraxella catarrhalis, Staphylococcus aureus, colonization, competition, children, polymicrobial, interference, upper respiratory tract infection, research

## Abstract

*Streptococcus pneumoniae, Haemophilus influenzae, Moraxella catarrhalis*, and *Staphylococcus aureus* often colonize the nasopharynx. Children are susceptible to bacterial infections during or soon after upper respiratory tract infection (URI). We describe colonization with these 4 bacteria species alone or in combination during URI. Data were from a prospective cohort of healthy children 6 to 36 months of age followed up for 1 year. Analyses of 968 swabs from 212 children indicated that *S. pneumoniae* colonization is negatively associated with colonization by *H. influenzae*. Competitive interactions shifted when *H. influenzae* and *M. catarrhalis* colonized together. In this situation, the likelihood of colonization with all 3 species is higher. Negative associations were identified between *S. pneumoniae* and *S. aureus* and between *H. influenzae* and *S. aureus*. Polymicrobial interactions differed by number and species of bacteria present. Antimicrobial therapy and vaccination strategies targeting specific bacterial species may alter the flora in unforeseen ways.

## Abstract 2

Competitive interactions between bacteria differ by number and species present; thus, vaccination and treatment strategies may alter nasopharyngeal flora and disease susceptibility.

*Streptococcus pneumoniae*, *Haemophilus influenzae*, *Moraxella catarrhalis*, and *Staphylococcus aureus* often asymptomatically colonize the nasopharynx of young children and are also associated with disease. *S. pneumoniae*, *H. influenzae*, and *M. catarrhalis* are the 3 most common otitis media pathogens (*1**,**2*). *S. pneumoniae* are also common causes of pneumonia, sepsis, and meningitis in young children (*3*). The proportion of young children colonized with any of these 3 bacteria species can be >50% in certain populations (*4**–**6*). *S. aureus* strains colonize up to 35% of young children and are associated with a wide range of diseases including soft tissue infections, sepsis, and pneumonia (*7**,**8*). Increases in the incidence of disease caused by community-acquired methicillin-resistant *S. aureus* are of great concern (*9*).

Host factors have been shown to influence colonization with *S. pneumoniae, H. influenzae, M. catarrhalis,* and *S. aureus*. These include host immunity, age, gender, race, out-of-home daycare, breastfeeding, and environmental exposure to tobacco smoke (*10*). The magnitude of host effects may differ by bacteria species.

Interactions between bacteria influence which species persist in the nasopharynx (*11**–**13*). Bacteria species may be positively associated; this occurs when they are found together more often than would be expected by chance. A negative association could occur when bacteria compete within same environment. Several studies have described a negative association between *S. pneumoniae* and *S. aureus* (*12**–**16*). Understanding of interactions between *S. pneumoniae*, *H. influenzae*, *M. catarrhalis,* and *S. aureus* is limited.

The nasopharyngeal flora change over time; the level of bacteria colonization is higher during upper respiratory infection (URI) (*6**,**17*). Knowledge is lacking regarding *S. pneumoniae*, *H. influenzae*, *M. catarrhalis,* and *S. aureus* interactions during URI because colonization studies either do not examine competitive interactions among all 4 pathogens or focus on healthy children (*5**,**11**,**16**,**18*). Children are susceptible to secondary bacterial infections during and after URI (*19*–*21*).

A better understanding of polymicrobial interactions in the nasopharynx is important for several reasons. Colonization is the initial step in the disease process (*22**, **23*). Colonized children serve as reservoirs for bacterial transmission to others in the community (*24*). Additionally, antibimicrobial drugs or vaccines, which target specific bacteria species, may alter polymicrobial interactions in the nasopharynx and have unanticipated consequences (*25**,**26*). The goals of our study were to 1) describe the prevalence of colonization with *S. pneumoniae, H. influenzae,* *M. catarrhalis*, and *S.* *aureus*; 2) evaluate interactions between *S. pneumoniae*, *H. influenzae, M. catarrhalis,* and *S. aureus*; and 3) estimate the effect of host factors on colonization with *S. pneumoniae*, *H. influenzae, M. catarrhalis*, and *S. aureus* after a URI in a prospective cohort of young children.

## Methods

### Study Design and Participants

We used data from a prospective study of otitis media complications of URI in children at the University of Texas Medical Branch (UTMB) at Galveston (*19**,**26*). The study was reviewed and approved by the UTMB Institutional Review Board. The parents of healthy children 6 months through 3 years of age, who were receiving medical care at UTMB from January 2003 through March 2007, were invited to enroll their children. Children with chronic medical problems and anatomic or physiologic defects of the ear or nasopharynx were excluded.

At enrollment, we collected information about demographic and URI risk factors. Parents were asked to describe their child’s race and ethnicity. We also obtained information regarding the number of weeks the child had been breastfed and the number of hours and days/week the child currently attended day care. We ascertained environmental exposure to tobacco smoke based on self-reports of whether any household members smoked cigarettes in the home.

The children in our study were followed up for 1 year. We requested that parents notify study staff when the child began to exhibit URI symptoms including nasal congestion, rhinorrhea, cough, sore throat, or fever. A study physician saw children as soon as possible after the onset of URI symptoms. At each study visit, the study physician obtained information regarding specific URI symptoms and examined the child’s ears. The children were then monitored closely for 3 weeks for the development of otitis media. The study physician collected a nasopharyngeal swab during the visit for each URI episode and when acute otitis media or sinusitis was diagnosed. URI episodes were categorized as the same episode if symptoms persisted. An episode of URI was considered new when symptoms of the previous episode subsided and the parents noted new symptoms of URI as described above. Given our prospective study design, many children had >1 URI episode and some had >1 visit/URI episode. We collected 1 swab/physician visit. Data regarding antimicrobial drug therapy during the past 7 days were collected by medical record review. A description of the methods is provided elsewhere (*19**,**26*).

A total of 294 children were enrolled in the original study (*19**,**26*). Included in these analyses are data from 212 (72%) children who experienced at least 1 URI, were seen by a study physician, and had a nasopharyngeal swab collected for bacterial culture. Thus, we excluded 82 children who did not have a URI and a swab for bacterial culture. Of these 82 children without URI visits, 35 (59%) were lost to follow-up in the first 6 months, 13 (38%) were lost to follow-up in months 7–11, and 34 (17%) completed 1 year of follow up.

Mini-Tip Culturette kits (Becton Dickinson Microbiology Systems, Cockeysville, MD, USA) were used for sample collection. Each swab was streaked onto 1 blood and 1 chocolate agar plate. We subcultured and identified suspected isolates of each species as follows: *S. pneumoniae* isolates were identified by using the optochin disk susceptibility test (Taxo P, Becton Dickinson Microbiology Systems), *H. influenzae* isolates were identified by the *Haemophilus* ID Quad Plate with Growth Factors (Becton Dickinson Microbiology Systems), *M. catarrhalis* isolates were identified by the API QuadFerm assay (bioMérieux, Inc., Hazelwood, MO, USA), and *S. aureus* isolates were identified by coagulase, catalase, and latex agglutination test (Staphaurex Plus, Remel, Lenexa, KS, USA).

### Statistical Methods

The main outcomes of interest were the relationships between bacteria during URI. All statistical analyses were conducted by using SAS version 9.1 (SAS Institute, Inc., Cary, NC, USA). We examined colonization by *S. pneumoniae*, *H. influenzae*, *M. catarrhalis,* and *S. aureus* by using repeated measures logistic regression with generalized estimating equations and an autoregressive correlation structure (AR1) using the procedure PROC GENMOD (SAS Institute, Inc.). Because each child could potentially have multiple URI episodes and contribute multiple bacterial swabs to the analysis, we used a repeated measures design to take into account variability of multiple samples from each child. To examine the effect of covariates on each bacteria species, we modeled colonization by *S. pneumoniae*, *H. influenzae*, and *M. catarrhalis* separately. We did not separately model the outcome of colonization by *S. aureus* because of low numbers of isolates obtained. Each model included the presence or absence of other bacteria species, as well as potential sampling-time confounders comprising time of swab collection after URI onset, antimicrobial drug therapy within the past 7 days, and age of the child at the time of swab collection. Host factors included in the model were gender, race, day care, breast-fed for >4 months, and environmental exposure to tobacco smoke.

## Results

Characteristics of the study participants are shown in Table 1. The median age of study participants was 12.0 months; mean age was 14.1 (SD 7.4) months. Most children were white, were cared for at home, and had not been breastfed for >4 months. Children were followed up for a median of 12 months and a mean of 10.7 (SD 2.8) months.

**Table 1:** Characteristics of study participants enrolled through the University of Texas Medical Branch, Galveston, Texas, USA, 2003–2007*

Characteristic	No. (%)
Age at enrollment, mo	
6–<12	92 (43.4)
12–<18	62 (29.2)
18–<24	30 (14.2)
24–<36	28 (13.2)
Gender	
F	103 (48.6)
M	109 (51.4)
Race	
White	124 (58.5)
Black	62 (29.2)
Asian	6 (2.8)
Other	20 (9.4)
Ethnicity	
Hispanic or Latino	95 (44.8)
Not Hispanic	117 (55.2)
Day care†	
No	147 (69.7)
Yes	64 (30.3)
Breast-fed for >4 mo	
No	173 (82.0)
Yes	38 (18.0)
Environmental exposure to tobacco smoke‡	
No	145 (68.4)
Yes	67 (31.6)

*Data given for 212 participants who experienced at least 1 upper respiratory infection, were seen by a study physician, and had a nasopharyngeal swab collected for bacterial culture. An additional 82 enrollees were excluded from the study because they did not experience an upper respiratory infection and did not have a nasopharyngeal swab collected for bacterial culture. Some numbers do not add up to 212 because of missing data. †No. hours and days/week in day care were grouped into any or none. Environmental exposure to tobacco smoke was based on parental self-report.

Individual children contributed between 1 and 20 swab specimens each (mean [SD] and median of 4.6 [3.8] and 3.0 swabs, respectively) from 1 to 18 URI episodes each (mean [SD] and median of 4.0 [3.3] and 3.0 episodes, respectively). Overall, at least 1 of the 4 species was isolated from 841 of 968 swab samples (86.9%) from 212 children. Of the 968 swabs, *S. pneumoniae* was present in 441 (45.6%)*, H. influenzae* was present in 314 (32.4%), and *M. catarrhalis* was the most common bacteria species identified in 611 (63.1%) swabs. *S. aureus* was relatively rare in comparison; 69 swabs (7.1%) were positive for this species. The distribution and colonization patterns of the 4 bacteria species by swab and number of URI visits are shown in Table 2.

**Table 2:** Distribution of bacteria on nasopharyngeal swabs collected from children with URI, University of Texas Medical Branch, Galveston, Texas, USA, 2003–2007*

Variable	Total no. (%) URI visits	No. (%) URI visits†				
		1	2	3–4	5–6	>6
Total no. patients	212	46 (21.7)	42 (19.8)	38 (17.9)	37 (17.4)	49 (23.1)
Total no. swabs	968	46 (4.8)	84 (8.7)	128 (13.2)	201 (20.8)	509 (52.6)
Bacteria present (% of no. of swabs in each visit category)						
0	127 (13.1)	9 (19.6)	9 (10.7)	13 (10.2)	19 (9.4)	77 (15.1)
1						
*Streptococcus pneumoniae*	79 (8.2)	1 ( 2.2)	9 (10.7)	15 (11.7)	20 (10.0)	34 (6.7)
*Haemophilus influenzae*	86 (8.9)	7 (15.2)	10 (11.9)	11 (8.6)	18 (9.0)	40 (7.9)
*Moraxella catarrhalis*	201 (20.8)	10 (21.7)	12 (14.3)	27 (21.1)	41 (20.4)	111 (21.8)
*Staphylococcus aureus*	24 (2.5)	1 (2.2)	2 (2.4)	2 (1.6)	3 (1.5)	16 (3.1)
2						
*S. pneumoniae*, *H. influenzae*	28 (2.9)	1 (2.2)	2 (2.4)	4 (3.1)	8 (4.0)	13 (2.6)
*S. pneumoniae*, *M. catarrhalis*	187 (19.3)	13 (28.3)	20 (23.8)	24 (18.8)	36 (17.9)	94 (18.5)
*S. pneumoniae*, *S. aureus*	8 (0.8)	0	1 (1.2)	1 (1.0)	4 (2.0)	2 (0.4)
*H. influenzae*, *M. catarrhalis*	67 (6.9)	2 (4.4)	5 (6.0)	7 (5.5)	13 (6.5)	40 (7.9)
*H. influenzae*, *S. aureus*	3 (0.3)	0	1 (1.2)	0	1 (0.5)	1 (0.2)
*M. catarrhalis*, *S. aureus*	17 (1.8)	0	2 (2.4)	3 (2.3)	2 (1.0)	10 (2.0)
3						
*S. pneumoniae, H. influenzae, M. catarrhalis*	124 (12.8)	2 (4.4)	8 (9.5)	19 (14.8)	31 (15.4)	64 (12.6)
*S. pneumoniae, H. influenzae, S. aureus*	2 (0.2)	0	1 (1.2)	0	0	1 (0.2)
*S. pneumoniae, M. catarrhalis, S. aureus*	11 (1.1)	0	1 (1.2)	2 (1.6)	4 (2.0)	4 (0.8)
*H. influenzae, M. catarrhalis, S. aureus*	2 (0.2)	0	0	0	1 (0.5)	1 (0.2)
4	2 (0.2)	0	1 (1.2)	0	0	1 (0.2)

*URI, upper respiratory tract infection. †Data are presented as no. of physician visits/child. Because of our prospective study design, many children had >1 URI episode during the follow-up period, and some had >1 physician visit/URI episode. One nasopharyngeal swab sample was taken at each physician visit.

Most swabs (849 [87.7%]) were collected within 7 days of URI onset; 119 (12.3%) were taken 8–30 days after URI onset. Of the 968 swab samples, only 54 (5.6%) were collected from children who had taken antimicrobial drugs within the past 7 days. Therefore, most swabs were collected from children who were not taking antimicrobial drugs at the time of swab collection (94.5%). Of the 212 children, 205 (>96%) had received at least 1 dose of the 7-valent pneumococcal conjugate vaccine (PCV7) at the time of enrollment. Most of the children had received all age-appropriate scheduled PCV7 vaccinations at their URI visit, 666 (69%) of samples were collected from children who had received the age-appropriate number of PCV7 doses at the time of swab collection. There was no association between being up to date with PCV7 vaccination and colonization with *S. pneumoniae* (p = 0.71). We did not further examine the effect of the pneumococcal vaccine further because of the high level of coverage in our study population.

Repeated measures logistic regression models predicting colonization by *S. pneumoniae, H. influenzae*, or *M. catarrhalis* are shown in Table 3. A positive association between bacteria is indicated by an odds ratio (OR) ≥1; a negative association is indicated by an OR <1. An OR of 1.0, or any 95% confidence interval that includes 1.0 indicates no significant association. The model predicting colonization by *S. pneumoniae* indicated that colonization by *H. influenzae* was negatively associated with *S. pneumoniae*. However, when *H. influenzae* and *M. catarrhalis* colonized together, they were positively associated with *S. pneumoniae* colonization. Colonization by *S. aureus* resulted in a 40% reduction in the odds of *S. pneumoniae* colonization. Older children were less likely to be colonized with *S. pneumoniae*; each 1-month increase in age was associated with a 2% decrease in the odds of *S. pneumoniae* colonization (Table 3). Antimicrobial drug therapy in the past 7 days was associated with decreased odds of *S. pneumoniae* colonization. The timing of swab collection after onset of URI symptoms and host characteristics such as gender, race, daycare, breastfeeding, and environmental exposure to tobacco smoke were not associated with colonization by *S. pneumoniae*.

**Table 3:** Predicted outcome of colonization with *Stretococcus pneumoniae*, *Haemophilus influenzae*, or *Moraxella catarrhalis* in young children after upper respiratory tract infection (968 swabs from 212 children; see Table 2)*

Parameters	OR (95% CI)		
	*S. pneumoniae*	*H. influenzae*	*M. catarrhalis*
*H. influenzae* x *M. catarrhalis* (p = 0.0003)†			
Neither (reference)	1.0	–	–
*H. influenzae* only	**0.59 (0.40–0.88)**	–	–
*M. catarrhalis* only	1.31 (0.95–1.81)	–	–
Both	**2.13 (1.35–3.38)**	–	–
*S. pneumoniae* x *M. catarrhalis* (p = 0.08)†			
Neither (reference)	–	1.0	–
*S. pneumoniae* only	–	**0.52 (0.32–0.83)**	–
*M. catarrhalis* only	–	**0.45 (0.29–0.69)**	–
Both	–	0.82 (0.52–1.30)	–
*H. influenzae* x *S. pneumoniae* (p<0.0001)†			
Neither (reference)	–	–	1.0
*H. influenzae* only	–	–	**0.44 (0.30–0.63)**
*S. pneumoniae* only	–	–	1.22 (0.88–1.70)
Both	–	–	**2.09 (1.30–3.37)**
*S. aureus*			
Absent (reference)	1.0	1.0	1.0
Present	**0.60 (0.36–0.99)**	**0.36 (0.17–0.76)**	0.72 (0.42–1.25)
Age (1-mo increase)‡	**0.98 (0.96–1.00)**	1.01 (0.98–1.03)	**0.98 (0.97–1.00)**
Antimicrobial drug therapy in past 7 days			
No (reference)	1.0	1.0	1.0
Yes	**0.40 (0.22–0.72)**	1.21 (0.69–2.13)	**0.52 (0.28–0.96)**
Time after URI onset, d			
<7 (reference)	1.0	1.0	1.0
>7	1.47 (0.96–2.27)	1.10 (0.70–1.73)	1.21 (0.81–1.80)
Gender			
F (reference)	1.0	1.0	1.0
M	1.05 (0.80–1.38)	**1.44 (1.08–.93)**	0.86 (0.65–1.14)
Race			
Not white (reference)	1.0	1.0	1.0
White	1.12 (0.84–1.48)	**0.42 (0.31–0.57)**	0.80 (0.60–1.07)
Day care			
No (reference)	1.0	1.0	1.0
Yes	1.32 (0.97–1.80)	**1.51 (1.09–2.09)**	1.09 (0.79–1.50)
Breast-fed >4 mo			
No (reference)	1.0	1.0	1.0
Yes	0.94 (0.69–1.29)	0.92 (0.65–1.29)	0.81 (0.59–1.12)
Environmental exposure to tobacco smoke			
No (reference)	1.0	1.0	1.0
Yes	1.13 (0.84–1.52)	0.93 (0.69–1.27)	0.91 (0.67–1.23)

*OR, odds ratio; CI, confidence interval. Significant ORs and 95% CIs are shown in **boldface**. Each model included variables representing presence or absence of other bacteria as well as all other variables listed. We did not model colonization of *S. aureus* because of low prevalence of this species (69/968 positive swabs). †p value from logistic regression model for overall significance of bacterial interaction. ‡Age (mo) of the child at the time of swab collection.

In our model examining *H. influenzae* colonization as the outcome, *H. influenzae* was negatively associated with *S. pneumoniae, M. catarrhalis*, and *S. aureus* (Table 3). In contrast to their association with *S. pneumoniae* colonization, age and antimicrobial drug therapy during the past 7 days were not significantly associated with colonization by *H. influenzae*. Host characteristics were associated with colonization by *H. influenzae*. Male gender and out-of-home daycare were associated with increased odds of *H. influenzae* colonization. White race was associated with decreased odds of *H. influenzae* colonization.

Our third model examined factors associated with colonization by *M. catarrhalis* (Table 3). *H. influenzae* was negatively associated with colonization by *M. catarrhalis*, but when *H. influenzae* and *S. pneumoniae* colonized together, they were positively associated with colonization by *M. catarrhalis*. Older children were less likely to be colonized with *M. catarrhalis*; each 1-month increase in age was associated with a 2% decrease in the odds of *M. catarrhalis* colonization (Table 3). Antimicrobial drug therapy in the past 7 days was associated with decreased odds of *M. catarrhalis* colonization. The timing of swab collection after onset of URI symptoms and host characteristics such as gender, race, daycare, breastfeeding, and environmental exposure to tobacco smoke were not associated with colonization by *M. catarrhalis*.

## Discussion

We describe nasopharyngeal colonization of children with *S. pneumoniae, H. influenzae, M. catarrhalis*, and *S. aureus* alone or in combination during URI. Our models predicting *S. pneumoniae* colonization indicated that *H. influenzae* is negatively associated with *S. pneumoniae*. However, when *H. influenzae* was present with *M. catarrhalis*, odds of *S. pneumoniae* colonization increased by >2-fold. Models predicting *H. influenzae* colonization indicated a negative association with *S. pneumoniae*, *M. catarrhalis*, and *S. aureus.* Competitive interactions between bacteria are complex after URI and may shift from negative to positive when additional bacteria species are present. Modeling *S. pneumoniae*, *H. influenzae,* and *M. catarrhalis* colonization separately showed that gender, race, and daycare were associated with colonization by *H. influenzae,* but not with colonization by either *S. pneumoniae* or *M. catarrhalis*.

Jacoby et al. used a multivariate random effects model to examine *S. pneumoniae* colonization in Aboriginal and non-Aboriginal children in Australia (*11*). Their study differed from ours in that they examined the relationship between *S. pneumoniae, H. influenzae*, *M. catarrhalis,* and *S. aureus* in pairwise combinations. These researchers also examined healthy children; we examined children who had a URI. Jacoby et al. observed positive associations between pairwise combinations of *S. pneumoniae* and *H. influenzae* and between *S. pneumoniae* and *M. catarrhalis*. They did not identify an association between *S. pneumoniae* and *S. aureus* or between *H. influenzae* and *S. aureus* (*11*).

Our results confirm a recent report describing a negative association between *H. influenzae* and *S. aureus* in HIV-negative children (*12*). Our data also support a growing body of literature describing negative associations between *S. pneumoniae* and *S. aureus* (*12**–**15*). For example, a cross-sectional study of 790 children younger than 40 months identified a negative association between *S. pneumoniae* colonization and *S. aureus* (OR 0.47; 95% confidence interval 0.28–0.78) (*13*).

An in vivo mouse model of competitive interactions between *S. pneumoniae* and *H. influenzae* has suggested mechanisms to explain our observations (*27*). Both *S. pneumoniae* and *H. influenzae* successfully colonized BALBc/SCID mice when each bacteria species was injected separately. However, *S. pneumoniae* was cleared rapidly when *H. influenzae* was present in a co-colonization model. The competitive interaction between *H. influenzae* and *S. pneumoniae* was dependent on complement and neutrophils (*27*). These researchers proposed that *H. influenzae* cellular components activate the host innate immune response, thus killing *S. pneumoniae* (*27*). *M. catarrhalis* was not examined in this model, but our data suggest that the additional presence of *M. catarrhalis* might alter the competitive balance between *S. pneumoniae* and *H. influenzae* and that all 3 bacteria species would successfully colonize.

In vitro studies have also demonstrated competition between *H. influenzae* and *S. pneumoniae* but predicted that *S. pneumoniae* should inhibit the growth of *H. influenzae*. Neuraminidase A is produced by *S. pneumoniae* and cleaves sialic acid*.* It has been shown to remove sialic acid from lipopolysaccharides of *H. influenzae* strains (*28*), potentially giving pneumococci a competitive advantage by making *H. influenzae* more susceptible to complement-mediated clearance. Furthermore, in vitro co-culture experiments indicate that *S. pneumoniae* can inhibit *H. influenzae* through the action of hydrogen peroxide (*29*). Interference between *S. pneumoniae* and *S. aureus* may also be caused by hydrogen peroxide production by *S. pneumoniae* (*30*).

Our results indicate that antimicrobial drug therapy in the past 7 days was associated with a lower prevalence of colonization with *S. pneumoniae* or *M. catarrhalis*. In contrast, antimicrobial drug therapy in the past 7 days was not associated with colonization by *H. influenzae*. Varon et al. studied the effect of antimicrobial drugs on colonization with *S. pneumoniae,* *H. influenzae*, and *M. catarrhalis* in a cohort of young children with URI (*31*). Children in this study received antimicrobial drugs for a mean treatment period of 8 days. Swab samples were taken before treatment and on days 2 through 6 after treatment. Results showed that colonization by *S. pneumoniae,* *H. influenzae*, and *M. catarrhalis* decreased after antimicrobial drug therapy (*31*). The magnitude of the effect differed by bacteria species and the specific antimicrobial drug prescribed. In general, antimicrobial drugs were less effective for reducing colonization with *H. influenzae* than with *S. pneumoniae* and *M. catarrhalis* (*31*).

The effect of age, gender, race, and breastfeeding on colonization differs by population studied (*10*). Daycare has consistently been associated with increased levels of colonization with *S. pneumoniae,* *H. influenzae*, and *M. catarrhalis* (*10*), as has exposure to other children in the household (*32**,**33*). Our study was limited by lack of data on age and number of siblings or other potential confounders such as household crowding and socioeconomic status.

Our study had additional limitations. A cross-sectional study of *S. aureus* and *S. pneumoniae* colonization indicated a negative association between PCV7 vaccine serotypes and *S. aureus* (*15*). No association was found between *S. pneumoniae* nonvaccine types and *S. aureus*. We were unable to examine the association between *S. pneumoniae* serotype and colonization. Along these lines, we did not have data regarding *H. influenzae* type B vaccination status and did not serotype our *H. influenzae* strains. Therefore, we were also unable to evaluate the effect of this vaccination on polymicrobial colonization.

Nasopharyngeal colonization likely involves a complex combination of factors including host characteristics that influence exposure to specific bacterial species, host immune responses that may result in killing the bacteria, and direct competitive interactions between bacteria species. In addition to the inhibiting effects of neuraminidase A and hydrogen peroxide already described, competitive interactions between bacteria may also include the secretion of small peptide inhibitors, competition for nutrients, and competition for receptor binding sites. It is also possible that the presence of 1 bacteria species could create a more hospitable niche for another bacteria species. We were unable to evaluate the precise molecular mechanisms that mediate these complex polymicrobial interactions, an important area for future research.

Our study had several strengths, including its longitudinal, prospective design. We examined nasopharyngeal carriage during URI, a time when children are at risk for secondary bacterial infections. In addition, we took advantage of repeated measures analytic techniques to examine microbe-level factors influencing bacterial colonization while controlling for host factors.

Results from our study have public health implications. Scientists have debated whether they should seek to eradicate disease by preventing nasopharyngeal colonization (*34*). Vaccines targeting nasopharyngeal carriage of *S. pneumoniae,* *H. influenzae*, and *M. catarrhalis* may be needed to prevent otitis media because simultaneous carriage of these 3 bacteria may increase risk for otitis media (*35*). Our data indicate that the elimination of nasopharyngeal colonization with bacteria such as *S. pneumoniae* and *H. influenzae* may increase risk for colonization with *S. aureus*. Scientists conducting a randomized trial of the effectiveness of pneumococcal vaccines noted an increase in *S. aureus* when spontaneously draining infected middle ears of vaccinated children (*25*). Factors that may increase the risk of colonization with *S. aureus* are of special concern given the spread of methicillin-resistant *S. aureus* (*9*). Researchers are attempting to develop an *S. pneumoniae* vaccine containing pneumococcal choline binding protein A, which would protect against sepsis and pneumonia without interfering with pneumococcal colonization (*36*). Although this type of vaccination strategy may eventually decrease the incidence of potentially fatal invasive pneumococcal disease, it is unlikely to prevent otitis media. Thus, the public health impact of a given intervention strategy may be hard to predict, and caution should be used when designing control strategies that target nasopharyngeal colonization.
